# MiR-124 inhibits the migration and invasion of ovarian cancer cells by targeting SphK1

**DOI:** 10.1186/1757-2215-6-84

**Published:** 2013-11-26

**Authors:** Hanwen Zhang, Qiuyu Wang, Qian Zhao, Wen Di

**Affiliations:** 1 Department of Obstetrics and Gynecology, Ren Ji Hospital, School of Medicine, Shanghai Jiao Tong University, Shanghai, 200127, China; 2 Department of Pathophysiology, Key Laboratory of Cell Differentiation and Apoptosis of National Ministry of Education, Shanghai Jiao Tong University School of Medicine (SJTU-SM), Shanghai, 200025, China; 3 Shanghai Key Laboratory of Gynecologic Oncology, Shanghai, 200127, China

**Keywords:** miR-124, SphK1, Migration, Invasion, Epithelial ovarian cancer

## Abstract

**Background:**

Epithelial ovarian cancer (EOC) is still a major gynecologic problem with poor 5 year survival rate due to distance metastases, despite routine surgery and chemotherapy. The precise underlying molecular mechanisms that trigger EOC migration and invasion are unclear. Recent studies suggest that the expression of microRNAs is widely dysregulated in ovarian cancer; and that they have evolved into tumorigenic processes, including cell proliferation, apoptosis and motility.

**Methods:**

The expression of miR-124 was assessed in clinical ovarian cancer specimens and cell lines using miRNA qRTPCR. The function of miR-124 on cell migration and invasion was confirmed *in vitro* through wound healing assay and transwell assay. Luciferase reporter assay was used to confirm target associations.

**Results:**

We showed that miR-124 is down-regulated in ovarian cancer specimens as well as in cell lines; and that low-level expression of miR-124 is much lower in highly metastatic ovarian cancer cells and tissues. Meantime, overexpression of miR-124 dramatically inhibits the motility of ovarian cancer cells *in vitro* and substantially suppresses the protein expression of SphK1, reported as an invasion and metastasis-related gene in human cancers, whose expression is markedly increased in both ovarian cancer cell lines and clinical samples, particularly in two highly metastasis cells, SKOV3-ip and HO8910pm as well as metastatic ovarian tumor tissues. Furthermore, SphK1 is identified as a direct target of miR-124, and knock-down of SphK1 in ovarian cancer cells, SKOV3-ip and HO8910pm, could mimic the inhibition of migration and invasion by miR-124, while re-introduction of SphK1 abrogates the suppression of motility and invasiveness induced by miR-124 in both cell lines.

**Conclusions:**

Our studies suggest a protective role of miR-124 in inhibition of migration and invasion in the molecular etiology of ovarian cancer, and a potentially novel application of miR-124 in the regulation of migration and invasion in EOC.

## Background

MicroRNAs (miRNAs) are endogenous non-coding small RNAs that execute post-transcriptional regulation by binding to the 3’-UTR of target genes [1-4], function as either oncogenes or tumor suppressor genes [5]. During recent years, more and more studies have reported a functional contribution of specific miRNAs in diverse biological processes [6-8], including deregulation of miRNAs by acting on their targeted genes in the progression and tumorgenesis of human cancers [5,9,10].

Ovarian cancer, one of the most common causes of death in women worldwide, is still a major problem in China over the past few decades [11-13] with high mortality, which is mainly due to the fact that more than 70% of patients are in late-stage, with distant metastases at the time of diagnosis [2,9]. It is reported that ovarian cancer is associated with multistep changes in the genome, in particular the expression and function of various microRNAs [9,14]. Although a large number of studies have shown a great potential for the use of microRNA in diagnosis, prognosis, and therapy in ovarian cancer [15,16], the precise association between microRNAs and migration and invasion of ovarian cancer is still relatively unclear.

MiRNAs are differentially expressed in ovarian cancer [2,10], including miR-124 [2]. MiR-124 was first reported to be highly expressed in neuronal cells [17], but its tumor-suppressor activity was significantly down-regulated in various cancer tissues [8,18-21]. Also, It has been reported that miR-124 involves in several malignant processes, including tumor proliferation, Epithelial-mesenchymal transition (EMT), and angiogenesis [6,19-23]. However, the expression level and the possible role of miR-124 in ovarian cancer remain to be explored.

SphK1 (Sphingosine kinase 1), a master kinase that regulates the balance between ceramide/sphingosine and S1P levels, mediates cellular behaviors and may determine cancer progression, including proliferation, migration, and invasion [24,25]. It has been demonstrated that SphK1 is an important enzyme encoded during neoplastic transformation [25,26]. In addition, SphK1 plays a critical role in motility and invasion of some cancer cells [27-29]. However, it is unclear whether SphK1 is responsible for malignant transformation of ovarian cancer.

We aimed to elucidate the involvement of miR-124 and SphK1 in migration and invasion of ovarian cancer. Our studies indicated that miR-124 was down-regulated in ovarian cancer cell lines and clinical samples. Interestingly, there is a significant correlation between the expression level of miR-124 and metastasis of ovarian cancer. On the other hand, our data showed that overexpression of miR-124 in ovarian cancer cells suppressed cell motility via SphK1, suggesting that SphK1 was identified as a direct and functional target for miR-124 in ovarian cancer progression. Thus, our findings provide valuable information toward unveiling the mechanisms of human ovarian cancer metastasis, and a novel target of potentially effective clinical therapies in the future.

## Methods

### Patients and ethics

Eleven malignant ovarian tumor tissues and normal ovarian tissues (as listed in Table 1) were selected from the archives of *the Department of Obstetrics & Gynecology at Ren Ji Hospital, Shanghai JiaoTong University School of Medicine* (Shanghai, China). This study was approved by *the Institutional Review Board of Ren Ji Hospital, Shanghai JiaoTong University School of Medicine*; and written informed consent was obtained from all patients. All clinical investigations were conducted according to the principles expressed in the Declaration of Helsinki.

**Table 1:** Clinical characteristics of women with ovarian cancer

**Patient no.**	**Age (year)**	**Grade**	**TNM**	**Stage**	**Ascites**	**Menopause**	**Histological subtype**	**Location**
1	51	3	T1cN0M0	Ic	+	No	Mucinous papillary adenocarcinoma	—
2	45	-	T1N1M0	IIIc	-	No	poorly differentiated carcinoma	Right
3	34	1	T1bN0M0	Ib	-	No	Mucinous cystadenocarcinoma	Right
4	62	2	T2N0M0	II	+	Yes	Adenocarcinoma	Left
5	60	3	T1aN0M1	IV	-	Yes	Serous papillary adenocarcinoma	Right
6	39	1	T1bN0M0	Ib	-	No	Mucinous cyst adenocarcinoma	Left
7	57	3	T1cN0M0	Ic	-	Yes	Serous adenocarcinoma	—
8	49	3	T2aN0M1	IV	+	Yes	Serous adenocarcinoma	Left
9	43	-	T2aN0M1	IV	+	No	poorly differentiated carcinoma	Right
10	73	-	T3cN1M1	IV	+	Yes	poorly differentiated carcinoma	Right
11	50	2	T3N1M0	IIIc	-	No	Serous adenocarcinoma	Left
12	47	-	-	-	-	-	-	Right
13	55	-	-	-	-	-	-	Right

### Cell lines and cell culture

Human ovarian SKOV3, HO8910pm cell lines with high-metastasis potential [30], HO8910, ES-2 and A2780 were obtained from Shanghai Institute of Cell Biology, China Academy of Sciences (Shanghai, China). OVCAR3 and OV90 were obtained from ATCC. SKOV3-ip1 and SKOV3-ip2 cells were selected from SKOV3 cell. All cells were cultured in RPMI-1640 medium supplemented with 10% fetal calf serum (GIBCO) in a humidified atmosphere of 5% CO_2_ at 37°C [31].

### Selection of invasive sublines from SKOV3 cell

To select a highly invasive subpopulation, SKOV3 cells were seeded in a corning 8 μm-pore transwell with Matrigel (BD Biosciences). After 24 hours, cells that had invaded to the other side of the transwell membrane were collected, expanded and then re-seeded into another Matrigel coated transwell. Such selection rounds for highly invasive cells were repeated two times.

### Quantitative PCR and immunoblotting

Total RNA was isolated using TRIzol reagent (Invitrogen, Carlsbad, CA, USA) according to the manufacturer’s instructions. Single-stranded cDNA was synthesized with the PrimeScript Reagent Kit (Promega, Madison, WI, USA). Real-time PCR was performed using SYBR Green PCR Master Mix (Applied Biosystems) on an ABI 7300HT real-time PCR system (Applied Biosystems, Foster City, CA, USA). Expression data were normalized to the internal control (U6) and the relative expression levels were evaluated using the Ct method. Primers for RT–PCR are listed in Table 2. For the protein expression analyses, standard Western blotting was carried out and the antibodies used were SphK1 (H00008877-M01, Abnova, Taiwan) and β-Actin (CP01, Calbiochem, MA, USA).

**Table 2:** Primers used for constructs and detection of the expression of miR-124.

**Name**	**Sequence (5’-3’)**	**Tm (°C)**	**Amplicon (bp)**
Sphk1-UTR-F	ACGCTCGAGGAATTGATGGTTAGCGAGGCC	64.5	1155
Sphk1-UTR-R	AGGGCGGCCGCTTATTTGGATTTGGTTCGTGGG	65	
Sphk1-UTR-Mut-site-F	GACCCCTGGGCCGCGCTGTCCGTAAGTGTCTACTTGCAGGACC	85	6658
Sphk1-UTR-Mut-site-R	GGTCCTGCAAGTAGACACTTACGGACAGCGCGGCCCAGGGGTC	84	
U6-F	CGCTTCGGCAGCACATATAC	59.4	64
U6-R	CAGGGGCCATGCTAATCTT	57.5	
MiR-124-F	GATACTCATAAGGCACGCGG	60.6	60
MiR-124-R	GTGCAGGGTCCGAGGT	57.9	
MiR-124-RT	GTCGTATCCAGTGCAGGGTCCGAGGTATTCGCACTGGATACGACGGCATTCT	87.8	

### Transfection of miR-124 or siRNA against SphK1

MiR-124 and the negative control were synthesized by Genepharma (Shanghai, China). Three siRNA duplex oligonucleotides fragments against SphK1 gene were synthesized by Ribobio (Guangzhou, China). Oligonucleotides were transfected with Lipofectamine 2000 reagent (Invitrogen, Paisley, Scotland, UK) at a concentration of 100 nM. For proliferation assays, cells were trypsinized 24 h after transfection. For migration, invasion, and western blot assays, cells were collected 48 h after transfection, as described previously.

### Monolayer wounding assay

For monolayer wounding assays, cells were plated in BD Falcon 24-well tissue culture plates after transfection and allowed to attach overnight. After serum deprivation for 12 h, confluent monolayers were scratched using a 10-μl pipette tip and washed once with serum-free medium. Twenty-four hours later, migration was assessed microscopically, and quantified using Multi Gauge V3.0 software (Fujifilm, Tokyo, Japan).

### In vitro cell migration and invasion assays

Cell migration was determined in Corning transwell insert chambers as described previously [31]. Cells suspended in 200-μl serum-free 1640 medium were placed into the upper chamber of the insert with or without Matrigel. After 24 h of incubation, cells remaining on the upper membrane were carefully removed. Cells that had migrated through the membrane were fixed with methanol and stained with 0.1% crystal violet (Biotime, China), imaged and counted using an IX71 inverted microscope (Olympus, Tokyo, Japan).

### Luciferase assays

HO8910pm and SKOV3-ip cells were plated into 24-well plates until 70% confluence before transfection. 100 ng wild-type or mutant SphK1 3’-UTR psiCHECK-2 plasmid (Promega, Madison, WI) was transiently co-transfected with 60 pmol miR-124 mimics or NC into HO8910pm and SKOV3-ip cells. Cell lysates were harvested 24 h after transfection and then firefly and *Renilla* luciferase activities were measured by the Dual-Luciferase Reporter Assay System (Promega, Madison, WI) on a Berthold AutoLumat LB9507 rack luminometer. *Renilla* luciferase activities were normalized to firefly luciferase activities to control for transfection efficiency.

### Statistical analysis

Data were expressed as the mean ± SD of at least three independent experiments. Group differences were compared using one-way ANOVA or two-tailed Student’s T-test from SPSS version 19.0 software (SPSS, Chicago, IL, USA). p value <0.05 was considered to be statistically significant.

## Results

### Reduced expression of miR-124 in highly metastatic ovarian cancer cell lines and clinical tumors

**Figure 1:**
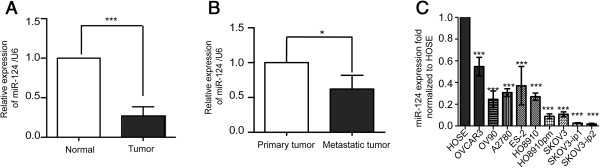
**Expression of miR-124 in cell lines and tissues of ovarian cancer. (A)** Expression of miR-124 in normal human ovarian tissues and ovarian tumors. **(B)** Comparison of miR-124 expression in five paired primary ovarian tumors and metastatic tissues. **(C)** The relative expression level of miR-124 in nine ovarian cancer cell lines. HOSE, the human normal ovarian epithelial cell line, was used as a control. Data are presented as means ± SD from three individual experiments. The symbols * and *** represent statistical significance at p < 0.05 and p < 0.001, respectively.

To determine the expression level of miR-124 in ovarian cancer progression, we first compared the expression levels between 13 clinical tumor samples and 2 normal ovarian tissues by stem-loop qRT-PCR. As shown in (Figure 1A and Table 1), the results showed that expression of miR-124 was decreased in 13 cases of ovarian cancer samples (p < 0.001), compared to normal ovarian tissues. Furthermore, we examined the expression of miR-124 between five paired metastatic and primary ovarian cancer tissues. Interestingly, we observed that the expression level of miR-124 was lower in metastatic tissues than in primary ovarian cancer samples (p < 0.05) (Figure 1B).

We further detected the abundance of miR-124 in nine ovarian cancer cell lines. Compared with HOSE (Pricells, Wuhan, China), a cell derived from human ovary surface epithelial cells, the expression of miR-124 evaluated by real time PCR in the above nine ovarian cancer cell lines were significantly reduced at different extent (Figure 1C), much lower in SKOV3-ip (Additional file 1) and HO8910pm [31] cells in particular. These results suggest that the expression of miR-124 is significantly decreased in human ovarian cancer specimens and cell lines, which may be involved in EOC metastasis.

### MiR-124 suppresses the migration and invasion of ovarian cancer cells in vitro

**Figure 2:**
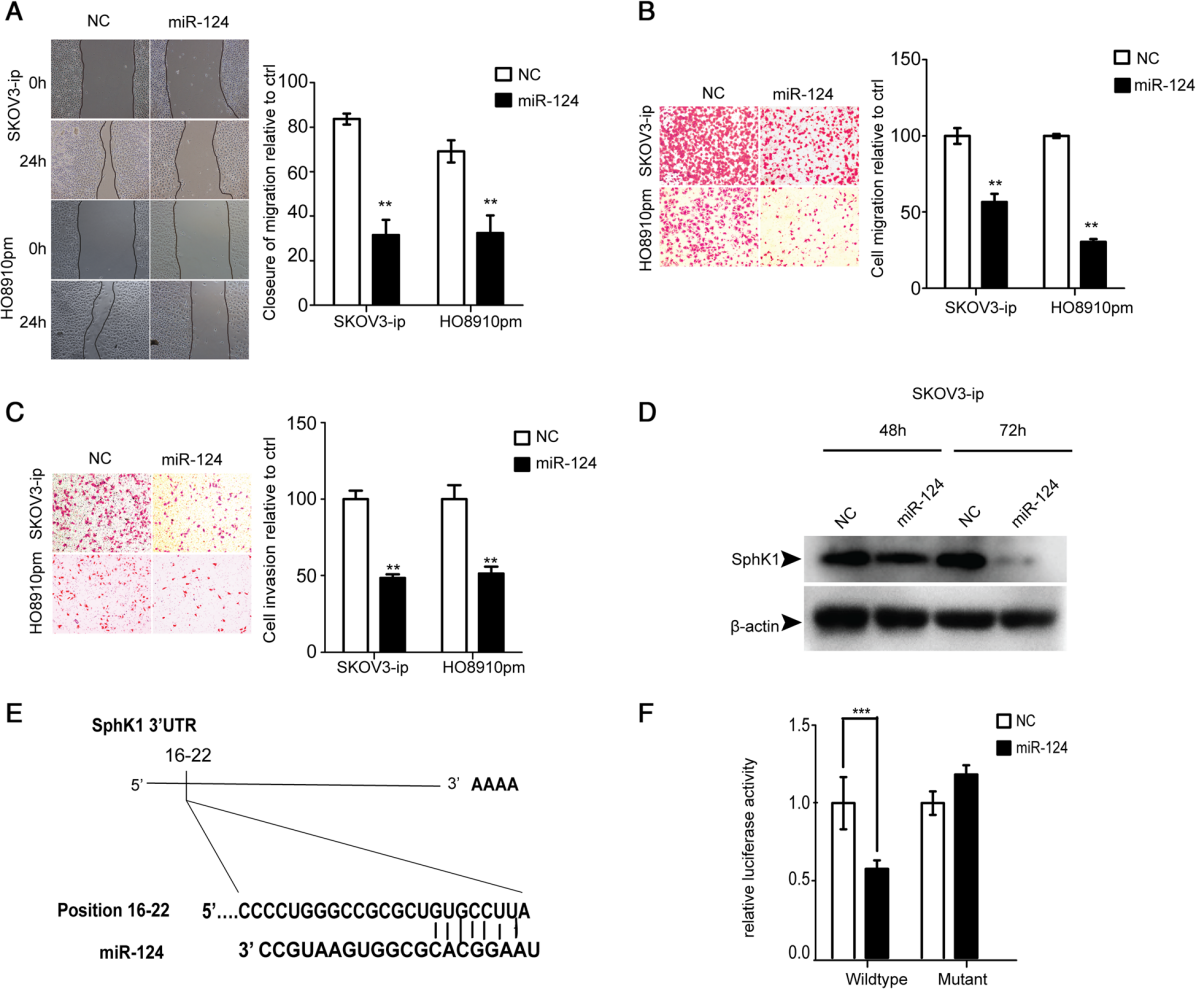
**MiR-124 directly targets SphK1 and inhibits the migration and invasion of ovarian cancer cells. (A)** Wound healing assay of SKOV3-ip and HO8910pm cells transfected with NC or miR-124 mimics. Representative pictures are shown at 0 and 24 h after the wound was made. **(B)** Transwell assay of SKOV3-ip and HO8910pm cells transfected with NC or miR-124 mimics. **(C)** Matrigel invasion assay of SKOV3-ip and HO8910pm cells transfected with miR-124 mimics or NC. **(D)** Expression of endogenous SphK1 in SKOV3-ip cells at 48 h and 72 h post transfection of miR-124 mimics or NC. β-actin was loaded as an internal control. **(E)** Diagram of the SphK1-3’-UTR with potential binding-sites for miR-124. **(F)** Relative luciferase activity of reporters including wild-type or mutant SphK1 3’-UTR co-transfected with NC or miR-124 mimics. ** and *** indicate significant difference at p < 0.01 and p < 0.001, respectively.

To investigate the functional role of miR-124 in epithelial ovarian cancer (EOC), we restored miR-124 expression in SKOV3-ip and HO8910pm cells, which shows the lowest expression of miR-124 in the nine ovarian cancer cell lines, by transient transfection with miR-124 mimics or negative control (NC). To verify the overexpression of miR-124, stem-loop qRT-PCR was performed and the results showed significantly increased expression of miR-124 in SKOV3-ip cells (Additional file 2A). In additionally, proliferation rate of cells post miR-124 transfection appeared no change (Additional file 3A, B). Given that SKOV3-ip and HO8910pm are highly metastatic cells, we then investigated the effect of miR-124 on their migration and invasion ability. The wound healing assay as well as migration Transwell assay indicated the ectopic expression of miR-124 can significantly inhibit cell migration compared to control group (Figure 2A, B). The same results were also observed in other two ovarian cancer cell lines, OV90 and OVCAR3 (Additional file 3C, D). Furthermore, invasion capability of SKOV3-ip and HO8910pm cells transfected with NC or miR-124 mimics were evaluated by Matrigel Invasion assay. As expected, the invasion ability of these two cells was decreased markedly as a result of overexpression of miR-124 (Figure 2C).

### SphK1 is a direct target of miR-124

It is well known that miRNAs regulate gene expression post transcriptionally by binding to the 3’-UTR of mRNAs. To verify the target involved in progression of ovarian cancer, we searched putative target genes via miRanda and TargetScan and we focused on SphK1 because of its rank and function associated with migration and invasion, particularly, the ectopic expression of miR-124 substantially decreased the expression of SphK1 in both SKOV3-ip and HO8910pm ovarian cancer cells assessed by western blot assay (Figure 2D and Additional file 2C), although real-time PCR analysis did not show obvious changes in mRNA expression of SphK1 (Additional file 2B). Besides, we also found that the ovarian cancer samples and metastatic ovarian cancer tissues showed a high level expression of SphK1 (Additional file 4A, B). Similarly, a much higher level expression of SphK1 is also observed in the SKOV3-ip and HO8910pm cells with high-metastasis potential showed (Additional file 4C, D). Collectively, the reduced miR-124 expression and overexpressed SphK1 was probably associated in ovarian cancer tissues and cells, which indicates that SphK1 was a direct target of miR-124.

In order to confirm that miR-124 directly targets SphK1, luciferase assay was performed. SKOV3-ip and HO8910pm cells were transfected with wild-type full length 3’-UTR of Sphk1 which was cloned into the psiCHECK™2 Vector as a control and mutant vector contained 4 mutated bases on only one biding site on SphK1-3’-UTR (Figure 2E and Additional file 2D upper panel), as well as miR-124 or NC. Overexpression of miR-124 significantly suppressed the luciferase activity of reporter genes containing 3’UTR of SphK1 compared with controls but partially rescued when the binding site was mutated (Figure 2F and Additional file 2D lower panel). The results are consistent with previous research [23], indicating that SphK1 is a predicted target of miR-124, and the inhibitory effect of miR-124 is due to direct interaction with the putative binding site of the 3’-UTR of SphK1.

### Knock-down of SphK1 blocks the migration and invasion of ovarian cancer cells

**Figure 3:**
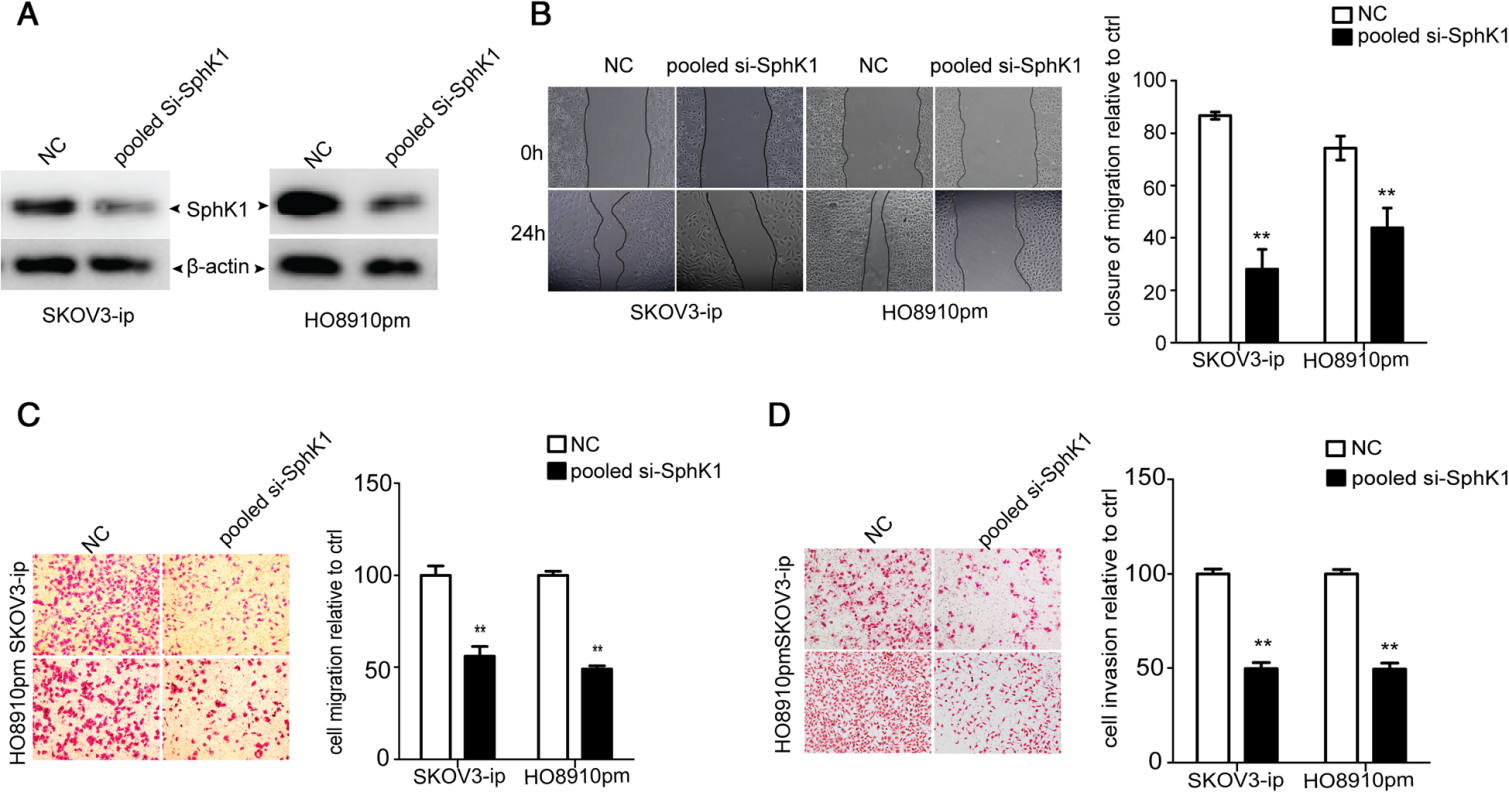
**SphK1 knockdown results in inhibition of migration and invasion in ovarian cancer cells. (A)** SphK1 detection in SKOV3-ip and HO8910pm cells transfected with NC or pooled si-SphK1. **(B)** Wound healing assay of SKOV3-ip and HO8910pm cells after transfected with NC or pooled si-SphK1. **(C)** Transwell assay of SKOV3-ip and HO8910pm cells subjected to NC or pooled si-SphK1. **(D)** Matrigel invasion assay of SKOV3-ip and HO8910pm cells after transfection with NC or pooled si-SphK1. Symbols ** and *** represent significance at p < 0.01 and p < 0.001, respectively.

To investigate whether MiR-124 has its inhibitory effect on migration and invasion of ovarian cancer through its target gene SphK1, we next determined to silence SphK1 and evaluated its expression by western blot in SKOV3-ip and HO8910pm cells. The silencing of SphK1 was confirmed by western blot (Figure 3A). In addition, knock-down of SphK1 did not affect the proliferation rate of SKOV3-ip and HO8910pm cells transfected with siRNA against SphK1 (Additional file 5A, B). Subsequently, we assayed migration and invasion ability of these two cells transfected with pooled siRNA fragments, migration and invasion were suppressed (Figure 3B, C and D). These results suggest that SphK1 participates in the motility of ovarian cancer cells.

### Expression of SphK1 reversed the miR-124-induced inhibition of cellular migration and invasion

**Figure 4:**
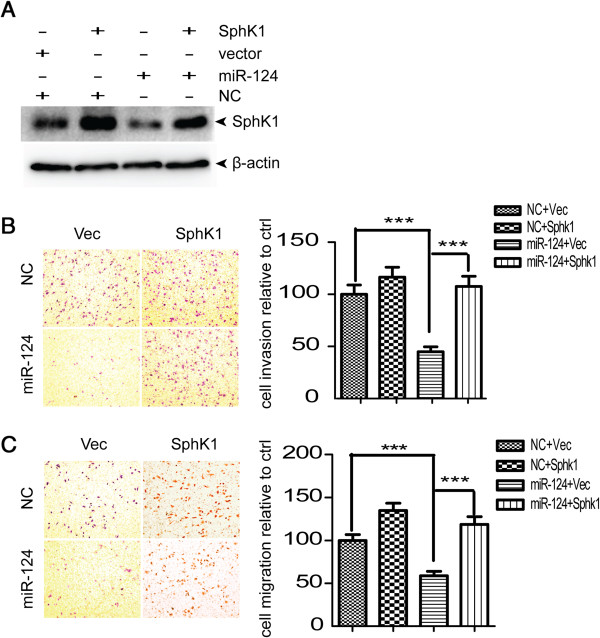
**Expression of SphK1 reversed the miR-124-induced inhibition of cellular migration and invasion. (A)** The transfection of pcDNA3.1 (−)–SphK1 restored the expression of Sphk1 in SKOV3-ip cells even with miR-124 co-transfection. **(B)** Transwell assay and **(C)** Matrigel invasion assay of SKOV3-ip cells after co-transfection of NC or miR-124 mimics together with either pcDNA3.1 (−)-vector or pcDNA3.1 (−)-SphK1. ***denotes statistical significance at p < 0.001.

In order to investigate the contribution of SphK1 to cellular migration and invasion, we ectopically expressed SphK1 together with miR-124 in SKOV3-ip cells to evaluate whether this may overcome the suppressive effect of miR-124 on cell migration and invasion. The SKOV3-ip cells were co-transfected with NC or miR-124 together with pCDNA3.1 (−)-vector or pCDNA3.1 (−)-SphK1 for 48 h. The expression of SphK1 recovered after SphK1 transfection (Figure 4A). Migration and invasion assays showed that enforced expression of SphK1 reversed miR-124-induced inhibition of migration and invasion (Figure 4B, C). Collectively, our results indicate that miR-124 participates in SphK1-mediated migration and invasion of EOC cells, suggesting that SphK1 is a functional mediator of miR-124 in EOC metastasis.

## Discussion

In the present study, we found that miR-124 was down-regulated in ovarian cancer cell lines and tumor tissues compared with normal ovarian surface epithelial cells and normal ovarian tissues. Furthermore, we showed that miR-124 inhibited EOC cell migration and invasion, which may be involved in the development of ovarian cancer metastasis. We also demonstrated that SphK1 is a direct target of miR-124 in EOC. Therefore, we now reasonably speculate that low expression of miR-124 contributes to SphK1-mediated migration in EOC cells.

Increasing evidence suggests to us that miRNAs are frequently dysregulated in various cancers, including ovarian cancer [3,5]. In this study, we observed that the expression level of miR-124 was low in ovarian cancer tissues, and even lower in the metastatic ovarian tissues. The abnormal expression of miRNAs in ovarian cancer has been previously evaluated [6,14,32]. In agreement with our results, Iorio et al. [2] have demonstrated that miR-124a is down-regulated in ovarian cancer tissues compared with normal ovarian samples. However, the role of miR-124 in ovarian cancer has not been reported in ovarian cancer [14,33]. Additionally, the directionality of expression of miR-124 (down-regulated) and SphK1 (up-regulated) that we observed appeared to be definitive in the two high-metastasis potential ovarian cancer cell lines, SKOV3-ip and HO8910pm. Also, clinical ovarian cancer samples were used to confirm the relationship between the endogenous expression levels of SphK1 and miR-124. In essence, this provided the possibility that the loss of miR-124 may lead to SphK1-mediated migration and invasion in ovarian cancer.

Invasion and metastasis are two leading attributes of malignant cancer [34] that result in high mortality in EOC. Our findings emphasize that the miRNA-induced down-regulation of genes may lead to the inhibition of migration and invasion of ovarian cancer cells, in agreement with several previous reports [20,21,35]. Although it has been reported that miR-124 is functionally involved in gynecological cancer [36], to the best of our knowledge, there are no published data on the role of miR-124 regarding migration and invasion in EOC. In this context, our study indicates that miR-124, by targeting SphK1, inhibits migration and invasion of ovarian cancer cells, suggesting that miR-124 plays a key role as a tumor suppressor in the motility of ovarian cancer cells; and that reduced expression of SphK1 contributes to distant metastases in EOC.

It is important to note that one microRNA can exert different functions by targeting multiple mRNAs[37]; that is, other genes regulated by miR-124 may also lead to ovarian carcinogenesis. Overexpression of miR-124 inhibits aggressiveness of hepatocellular carcinoma cell by targeting ROCK2 and EZH2[21]. Additionally, Fowler et al. reported that IQGAP1, laminin c1 and integrin b1 (which are not the only 3 targets of miR-124) are also associated with migration and invasion in clinical glioblastoma specimens, compared with normal brain tissue [38]. In addition, We confirmed through luciferase reporter gene assays that miR-124 directly targets SphK1 by binding the 3’-UTR of SphK1 mRNA, which is consistent with Xia et al. [23]. MiR-124 blocks migration and invasion of ovarian cancer cells by targeting SphK1, which would constitute a promising target for rational cancer therapy. Also, our results provided another mechanism for modulating the protein expression of SphK1 in ovarian cancer cells. Thus, it is possible that miR-124 could attenuate EOC invasion partly through inhibition of the SphK1pathway.

## Conclusions

In conclusion, our current study provides novel evidence that ectopic expression of miR-124 significantly suppresses migration and invasion of ovarian cancer cells and down-regulates SphK1, which is a direct functional target of miR-124. The loss of miR-124 may then contribute to the migration and invasion of EOC cells. The newly identified miR-124/SphK1 link provides novel insight into the metastasis of EOC, especially with respect to invasion and metastasis *in vitro*; and represents a new potential therapeutic target for the treatment of EOC.

## Abbreviations

EOC: Epithelial ovarian cancer; MiRNA: microRNA; EMT: Epithelial-mesenchymal transition; SphK1: Sphingosine kinase 1.

## Competing interests

The authors declare no competing interests.

## Authors’ contributions

HZ and QW performed the experiments, prepared the data and drafted the manuscript. QZ and WD are co-mentors, provided input of studies and edited the manuscript. All authors read and approved the final manuscript.

## Supplementary Material

Additional file 1 **Ovarian cancer subline SKOV3-ip with high metastatic capacity.** Matrigel invasion assay showed the invasive ability of was about 4-fold greater than the parent cell line SKOV3 (P < 0.05). Click here for file

Additional file 2 **Validation of miR-124 targeting SphK1 in HO8910pm cells.** (A) Detection of miR-124 expression in SKOV3-ip cells after transfection with NC or miR-124 mimics. (B) Ectopic expression of miR-124 does not affect SphK1 mRNA in SKOV3-ip cells. (C) Immunoblotting of endogenous SphK1 expression in HO8910pm cells transfected with NC or miR-124 mimics. (D) Upper panel: Schematic representation of the luciferase reporter constructs. Lower panel: luciferase reporter assay performed in HO8910pm cells. Click here for file

Additional file 3 **Overexpression of miR-124 has no effects on proliferation but suppresses the motility of cells.** (A), (B) miR-124 overexpression does not affect cell proliferation of SKOV3-ip and HO8910pm cells. (C) Transwell migration assay of OV90 and OVCAR3 cells transfected with NC or miR-124 mimics. (D) Matrigel invasion assay of OV90 and OVCAR3 cells transfected with NC or miR-124 mimics. Both cell lines transfected with 100 nM miR-124 mimics or NC were inoculated in 6- well plates at 24 h. CCK-8 assay was performed at 48 h and 72 h after transfection. Click here for file

Additional file 4 **Expression of SphK1 in ovarian cancer cell lines and clinical ovarian cancer samples.** (A) Expression levels of SphK1 in normal tissue and ovarian cancer tissues. (B) Comparison of SphK1expression in five paired primary ovarian tumors and metastatic tissues. (C) Expression of SphK1 in the human normal ovarian epithelial cell line (HOSE) and nine ovarian cancer cell lines. (D) Western blot of SphK1 protein expression in nine ovarian cancer cells. Click here for file

Additional file 5 **Knock-down SphK1 has no effects on proliferation of SKOV3-ip and HO8910pm cells.** (A), (B) pooled Si-SphK1transfection does not affect cell proliferation of SKOV3-ip and HO8910pm cells. Click here for file
