# Supplementary figures and images for: MiR-124 inhibits the migration and invasion of ovarian cancer cells by targeting SphK1
Source: J Ovarian Res. 2013 Nov 26;6:84. doi: 10.1186/1757-2215-6-84 (PMC3879084; doi:10.1186/1757-2215-6-84)

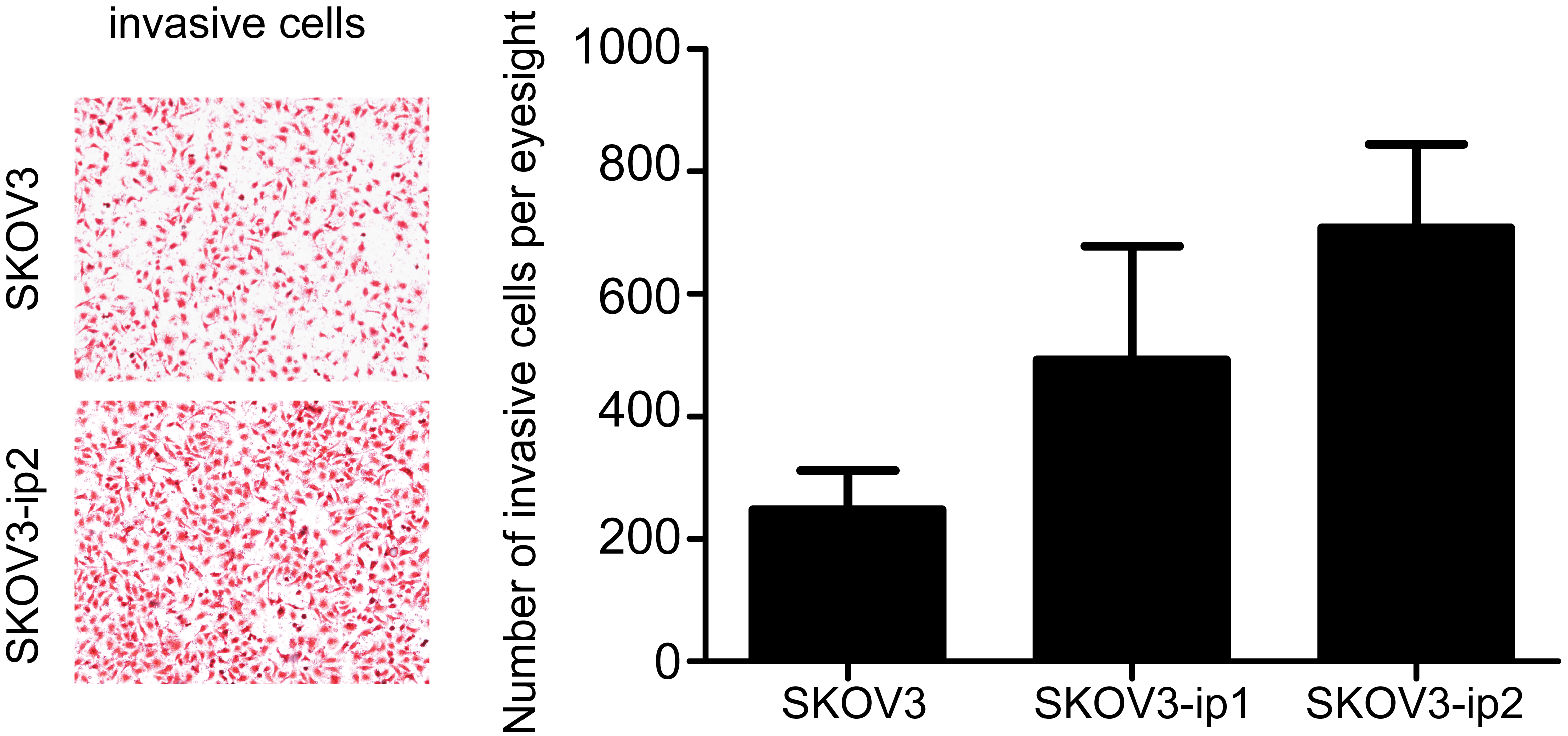

Supplement: Additional file 1 — Ovarian cancer subline SKOV3-ip with high metastatic capacity. Matrigel invasion assay showed the invasive ability of was about 4-fold greater than the parent cell line SKOV3 (P < 0.05). [file 1757-2215-6-84-S1.tiff]

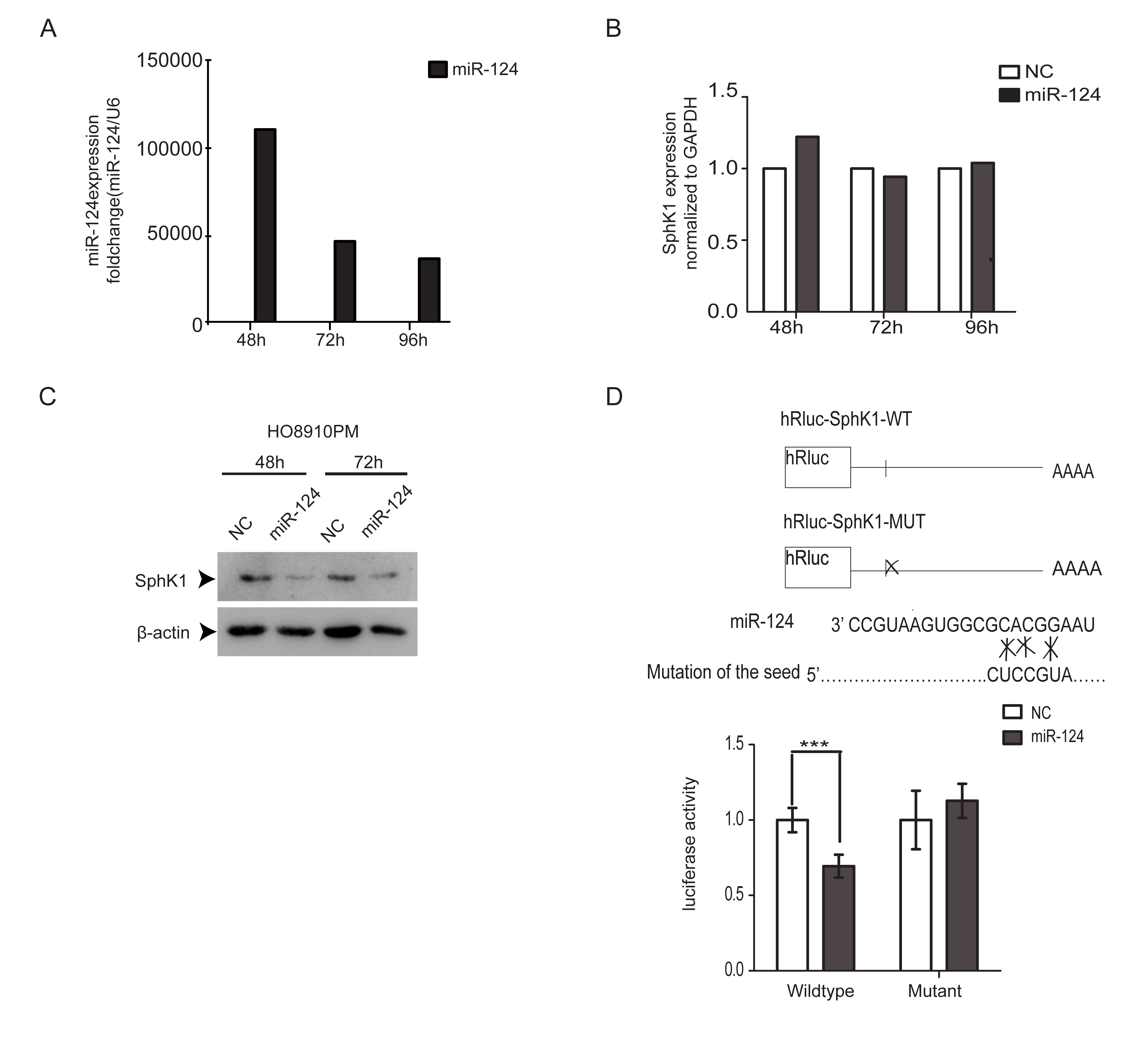

Supplement: Additional file 2 — Validation of miR-124 targeting SphK1 in HO8910pm cells. (A) Detection of miR-124 expression in SKOV3-ip cells after transfection with NC or miR-124 mimics. (B) Ectopic expression of miR-124 does not affect SphK1 mRNA in SKOV3-ip cells. (C) Immunoblotting of endogenous SphK1 expression in HO8910pm cells transfected with NC or miR-124 mimics. (D) Upper panel: Schematic representation of the luciferase reporter constructs. Lower panel: luciferase reporter assay performed in HO8910pm cells. [file 1757-2215-6-84-S2.tiff]

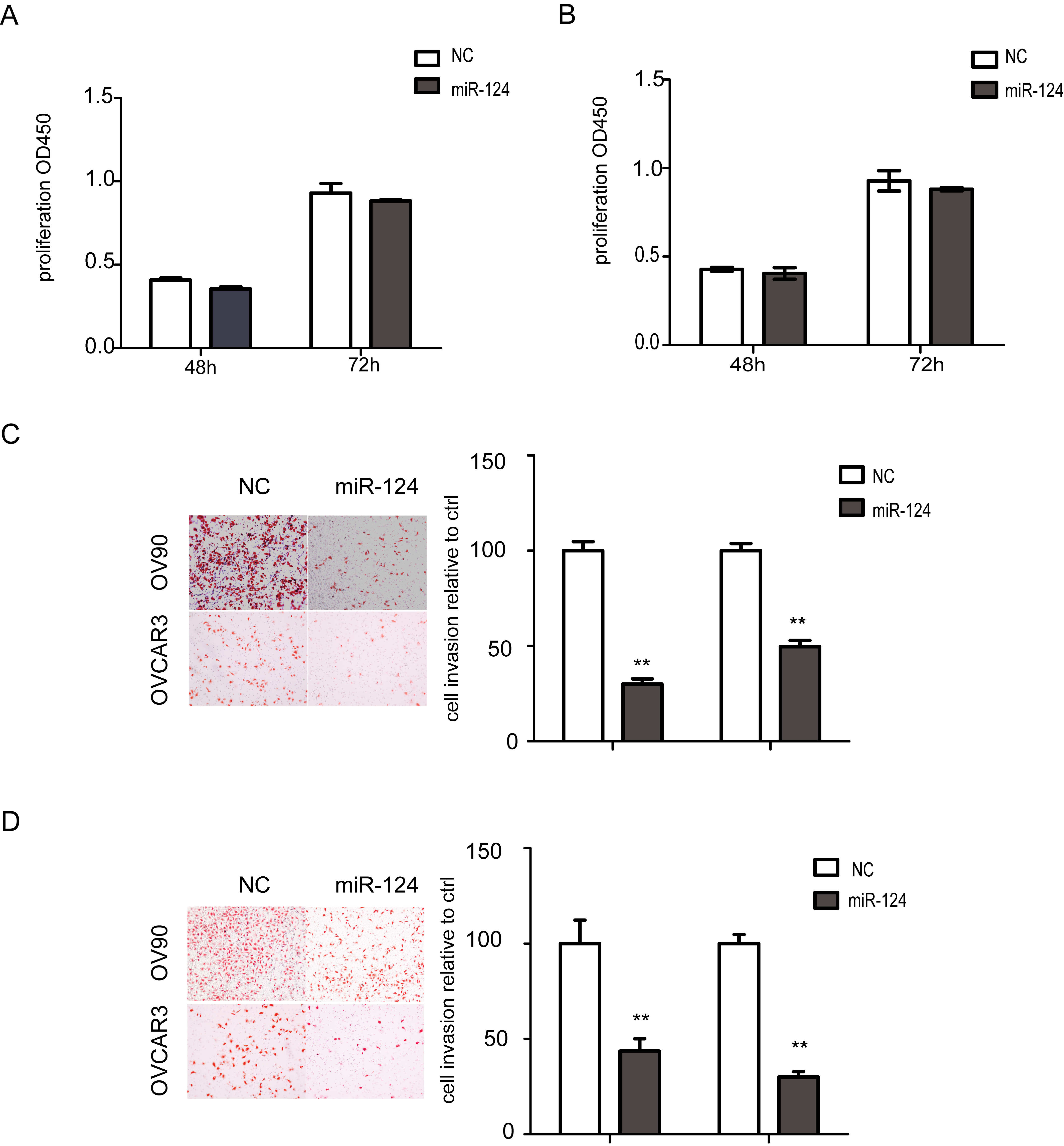

Supplement: Additional file 3 — Overexpression of miR-124 has no effects on proliferation but suppresses the motility of cells. (A), (B) miR-124 overexpression does not affect cell proliferation of SKOV3-ip and HO8910pm cells. (C) Transwell migration assay of OV90 and OVCAR3 cells transfected with NC or miR-124 mimics. (D) Matrigel invasion assay of OV90 and OVCAR3 cells transfected with NC or miR-124 mimics. Both cell lines transfected with 100 nM miR-124 mimics or NC were inoculated in 6- well plates at 24 h. CCK-8 assay was performed at 48 h and 72 h after transfection. [file 1757-2215-6-84-S3.tiff]

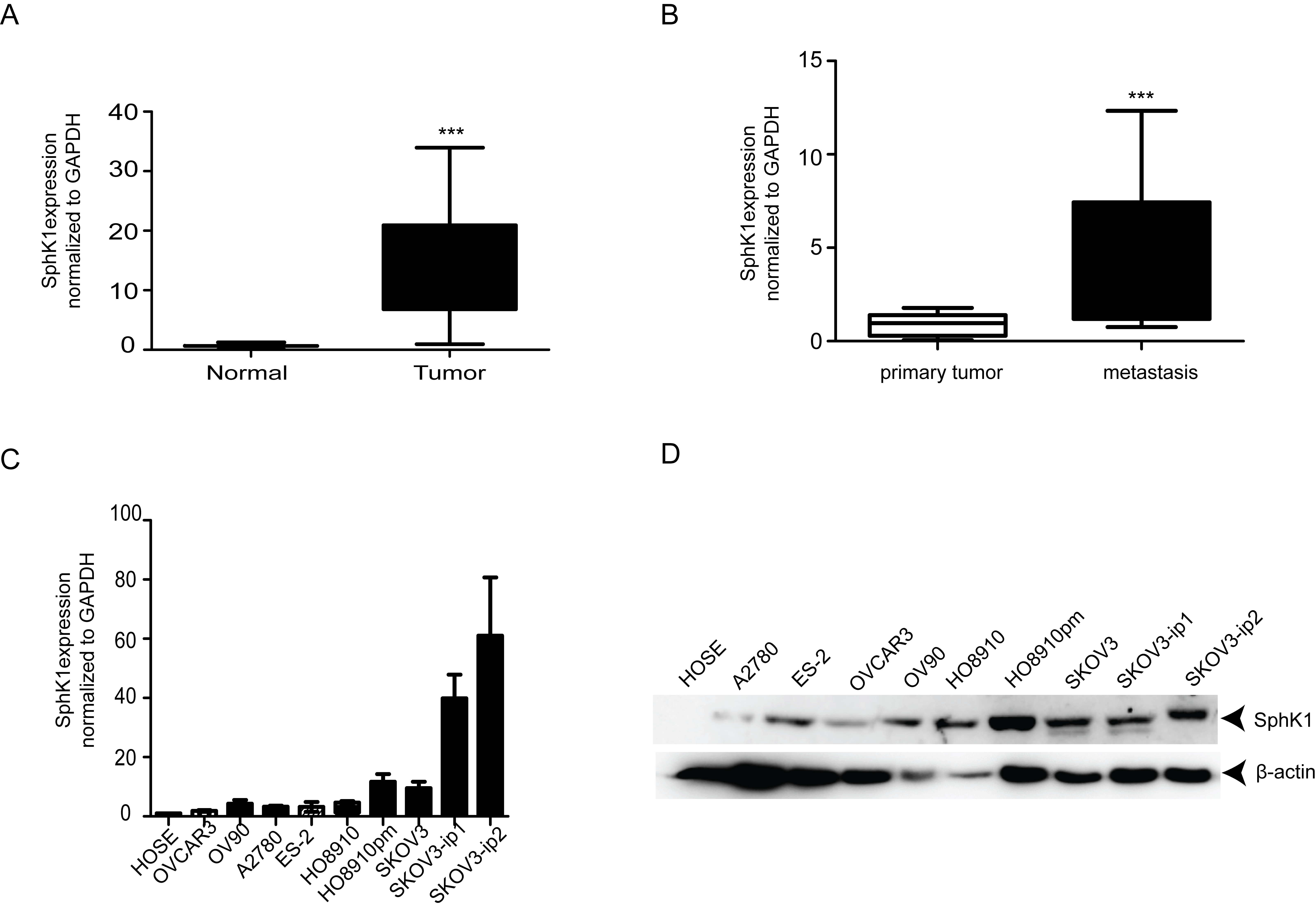

Supplement: Additional file 4 — Expression of SphK1 in ovarian cancer cell lines and clinical ovarian cancer samples. (A) Expression levels of SphK1 in normal tissue and ovarian cancer tissues. (B) Comparison of SphK1expression in five paired primary ovarian tumors and metastatic tissues. (C) Expression of SphK1 in the human normal ovarian epithelial cell line (HOSE) and nine ovarian cancer cell lines. (D) Western blot of SphK1 protein expression in nine ovarian cancer cells. [file 1757-2215-6-84-S4.tiff]

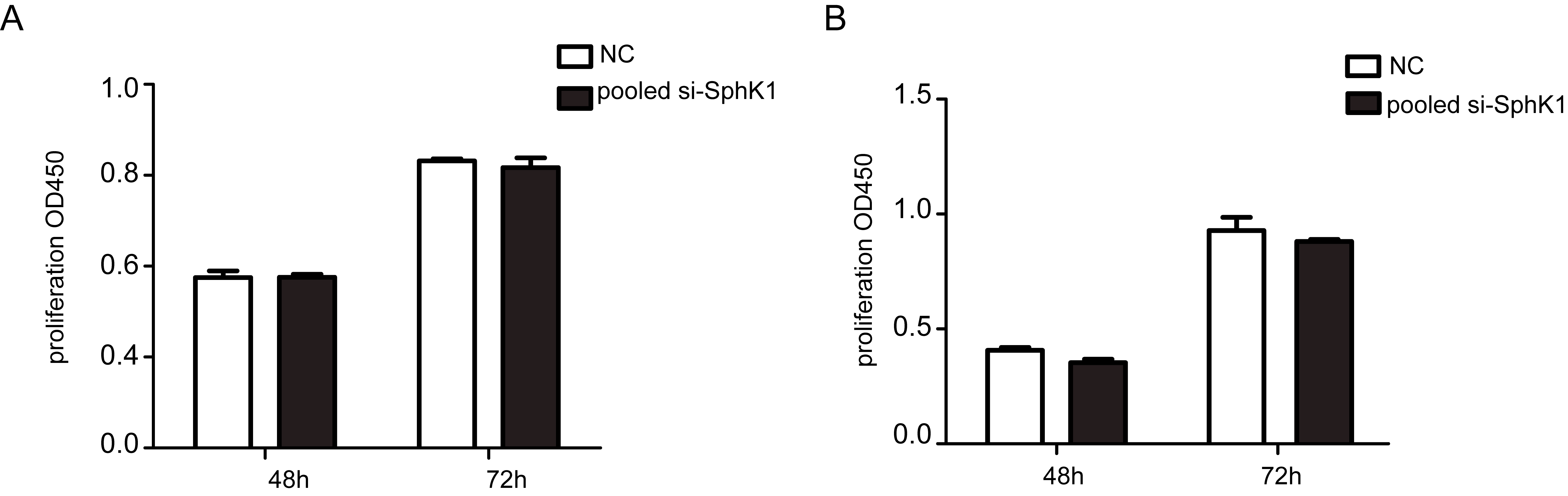

Supplement: Additional file 5 — Knock-down SphK1 has no effects on proliferation of SKOV3-ip and HO8910pm cells. (A), (B) pooled Si-SphK1transfection does not affect cell proliferation of SKOV3-ip and HO8910pm cells. [file 1757-2215-6-84-S5.tiff]
